# Mental health and behaviour of students of public health and their correlation with social support: a cross-sectional study

**DOI:** 10.1186/1471-2458-11-871

**Published:** 2011-11-16

**Authors:** Éva Bíró, Róza Ádány, Karolina Kósa

**Affiliations:** 1 Division of Health Promotion, Department of Preventive Medicine, Faculty of Public Health, Medical and Health Science Centre, University of Debrecen, Debrecen, Hungary; 2 Department of Preventive Medicine, Faculty of Public Health, Medical and Health Science Centre, University of Debrecen, Debrecen, Hungary; 3 4012 Debrecen, Hungary, POB. 2

## Abstract

**Background:**

Future public health professionals are especially important among students partly because their credibility in light of their professional messages and activities will be tested daily by their clients; and partly because health professionals' own lifestyle habits influence their attitudes and professional activities. A better understanding of public health students' health and its determinants is necessary for improving counselling services and tailoring them to demand. Our aim was to survey public health students' health status and behaviour with a focus on mental health.

**Methods:**

A cross-sectional study was carried out among public health students at 1-5-years (*N *= 194) with a self-administered questionnaire that included standardized items on demographic data, mental wellbeing characterized by sense of coherence (SoC) and psychological morbidity, as well as health behaviour and social support. Correlations between social support and the variables for mental health, health status and health behaviour were characterized by pairwise correlation.

**Results:**

The response rate was 75% and represented students by study year, sex and age in the Faculty. Nearly half of the students were non-smokers, more than one quarter smoked daily. Almost one-fifth of the students suffered from notable psychological distress. The proportion of these students decreased from year 1 to 5. The mean score for SoC was 60.1 and showed an increasing trend during the academic years. 29% of the students lacked social support from their student peers. Significant positive correlation was revealed between social support and variables for mental health. Psychological distress was greater among female public health students than in the same age female group of the general population; whereas the lack of social support was a more prevalent problem among male students.

**Conclusions:**

Health status and behaviour of public health students is similar to their non-students peers except for their worse mental health. Future public health professionals should be better prepared for coping with the challenges they face during their studies. Universities must facilitate this process by providing helping services targeted at those with highest risk, and developing training to improve coping skills. Social support is also a potentially amenable determinant of mental health during higher education.

## Background

Young adulthood entails remarkable transitions the most important of which is that from studying to employment. This period may have implications for health as lifestyle and habits change for the better or for the worse [1]. Considering the pattern of young adults' health, neuropsychiatric disorders are the main cause of burden in high-income countries, especially in those aged 15-24 years [2]. The critical importance of mental health of students is reflected by an increasing number of university and college students seeking counselling services for psychological problems including learning disabilities, self-injuries, eating disorders, alcohol problems, illicit drug use, concerns of sexual assault on campus, and problems related to earlier sexual abuse in the USA during the last decade. Approximately one-fifth of counselling centre clients had severe psychological problems. Ninety-four percent of directors also noted an increase in the number of students seeking counselling who had already been taking psychiatric medication [3]. Twenty-eight percent of freshman polled in a national survey reported feeling frequently overwhelmed, and 8% reported feeling depressed in the USA. A longitudinal study of psychological distress in college found that although distress levels peaked during the first year and then declined for most students, and a subset of students manifested severe, chronic levels of distress that did not decrease over time [4-6].

Lived values, among others, are key determinants of mental health according to the World Federation for Mental Health [7]. Given the societal difference of values among post-socialist countries and established market economies [8] in addition to the complex issues of young adulthood and its implications for long-term health, monitoring the health of college students in various countries may help uncover problems early on, and facilitate the provision of services or interventions to cope with these problems. For one, mental health problems, if left unrecognized and untreated, may lead students to drop out or fail their studies, attempt or commit suicide, or engage in other risky, dangerous behaviours that may result in serious injury, disability, or death [9]. For the other, it has been shown that health professionals' own lifestyle habits can influence their attitudes and health education practices with patients [10]. However, many health workers have a long way to go towards the lifestyle they preach to their clients [11].

It is of utmost importance that health workers, among them public health professionals form their lifestyle preferably during their studies so that it is conducive to health and enables them to appear as credible sources of information in their employment. While a number of studies have been published on the worrysome health and health risks of medical students, especially in terms of high levels of stress during their training [12-14] public health students have been out of sight in this respect.

Mental health status can be measured by a host of tools. Some of them are more appropriate for measuring short-term changes such as the widely used General Health Questionnaire (GHQ), an extensively used screening instrument of varying length that is appropriate for detecting non-psychotic psychological morbidity including anxiety and depression in the general population [15]. Other tools can be used to measure constructs that are more stable in time and characteristic of the person, such as sense of coherence (SoC) defined by A. Antonovsky [16]. Sense of coherence is a global orientation expressing a pervasive, enduring and dynamic feeling of confidence that reflects a person's view of life and also his/her capacity to respond to stressful situations. SoC reflects strain resistance resources and how these resources are used to maintain and develop health [16].

Social capital has been shown to be a major determinant of health, and its strengthening has salutogenic effects. As to its measurement, two schools of thought can be distinguished: the social cohesion theory holds social capital as a group attribute, whereas the network theory considers it an attribute measurable at the individual and group levels as well [17]. Social support is an individually measurable dimension of social capital according to the network theory of social capital, defined as information leading the subject to believe that he is cared for and loved, esteemed, and a member of a network of mutual obligations [18].

Our goal was to survey public health students' mental health and health behaviour during the full course of training of 5 years in Hungary. Our research model was built on the biopsychosocial model of health defining age and sex as biological determinants; mental health was measured by sense of coherence and psychological distress, while social capital approximated by social support was considered as a social determinant of health.

## Methods

### Study population

A cross-sectional study was carried out among public health students of years 1-5 at the Faculty of Public Health of the University of Debrecen, Hungary in 2008 (students at 4-5 years started their studies before public health studies were split into bachelor and master degrees). 150 students studied for a bachelor degree in the Bologna scheme and 44 students were still in the pre-Bologna training lasting for 5 years. At that time, only this university in Hungary provided 5-year training in public health. Of the 194 students from first to fifth year, 149 were present at the time of data collection. All but 3 of the completed questionnaires were eligible for evaluation (the overall response rate was 75%; response rates by study year: 1st year 65.5%, 2nd year 71%, 3rd year 86.5%, 4th year 80.5%; 5th year 100%).

**Figure 1:**
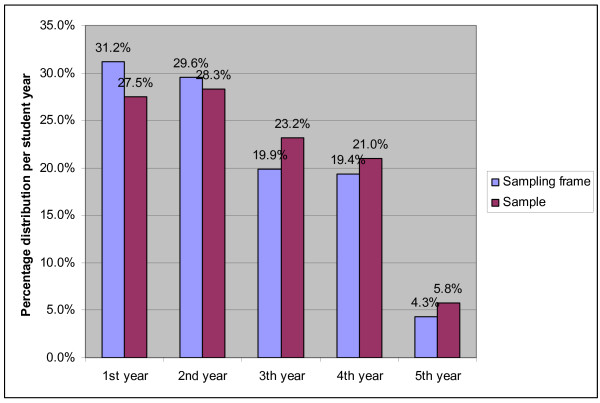
**Distribution of the sample and sampling frame by study year**.

16.5% of the students were male and 83.5% were female, constituting a representative sample by sex of public health students (17.5% male, 82.5% female, p: 0.809). Representation by study year is shown in Figure 1. Mean age in the sample was 20.6 years (18-25 years, standard deviation, SD: 1.53), that did not differ significantly from the mean age of all students at the Faculty (20.6 years, 18-26 years, SD: 1.33; p: 0.749). Two third of the students were between 19 and 21 years of age.

### Data collection

Each student was invited in person after class to fill a paper-based, self-administered, anonymous questionnaire. The research was carried out in compliance with the Helsinki Declaration. Ethical permission for the study (DEOEC RKEB/IKEB: 2506-2006) was issued by the Commission on Research Ethics of the Medical and Health Science Centre of the University of Debrecen, Hungary. The students were informed in writing and in person that participation was voluntary and they had the right to refuse to participate. No consent form was requested to be signed as no personal data were collected. To avoid pressuring for participation, student volunteers were asked to distribute and collect the questionnaires.

### Questionnaire domains

The questionnaire used in this survey was identical to that used in a previous survey among medical students [19] and included scales on mental health (sense of coherence, psychological morbidity) perceived health, demographic (age, sex, residence) and socioeconomic (parents' educational level, family's economic status) data, social support, as well as health behaviour: physical activity, diet, body weight & height, sexual behaviour, smoking, alcohol & drug use. Items on substance use were adapted from the questionnaire of the European School Survey Project on Alcohol and Other Drugs (ESPAD) [20,21]. Items not referred separately were taken from the tool of the Hungarian National Health Interview Survey (HNHIS) of 2003 [22].

#### Social support

Social support was measured by the Hungarian version of the Health and Lifestyle Survey and Health Survey for England [23]. Briefly, respondents answered seven questions and scores of 1-3 were obtained for each question and overall scores ranged from 7 to 21. The maximum score of 21 indicated no lack of social support, scores of 18 to 20 indicated a moderate lack of social support and scores of 17 showed that individuals perceived a severe lack of social support [24].

#### Sense of coherence (SoC-13)

The validated Hungarian version [25] of the abbreviated (13 items) form was used in the present survey. Items are answerable on a Likert scale from 1 to 7, total scores vary between 13 and 91. A higher score indicates stronger SoC.

#### Psychological distress: general health questionnaire (GHQ-12)

The short (12-item) version of GHQ has been used to detect psychological morbidity. Its use above the age of 17 years has been well established [26]. Answers are given on a Likert scale from 1 to 4. Cases are detected by scoring in the simplest manner [27], which designates each symptom as absent or present according to the usual (0-0-1-1) method. Therefore scores ranged between 0 and 12. The threshold indicating notable psychological distress (score above 4) was identical to that used in the Hungarian National Health Interview Survey of 2003 [28] in order to make the comparison of the two datasets possible.

### Statistical methods

Questionnaires were coded by study years. Intercooled Stata 9.0 for Windows was used for data analysis. For sense of coherence, psychological distress (GHQ), and social support, a total score was calculated and used for analysis as it is described under "Questionnaire domains". Social support was categorized as severely or moderately lacking or sufficient. Correlation between variables for mental health, health status and health behaviour were characterized by pairwise correlation, the significance level was set at 0.01.

Results were compared with results of the general population of the same age from the Hungarian National Health Interview Survey of 2003. We used the two-sample unpaired *t *test to compare means and the two-sample test of proportion to compare proportions for which the significance level was set at 0.05.

## Results

### Basic demographics

Nearly one third (32%) of the respondents had mothers with college degrees for highest education, 42% of the students had high school graduate mothers, 18% of them had mothers with vocational training, and the mothers of 8% completed only elementary school. Less than one fifth (18.5%) of the students had college-educated fathers, and in nearly equal proportion (38%-40%) high school graduate fathers and fathers with vocational training, whereas 1.5% of fathers finished elementary school (2% did not know their fathers' qualification). Almost two fifth (41%) of the students thought that economic status of their family was good or very good, 47.5% ranked it as satisfactory, and 11.5% as bad or very bad.

### Health status

More than two third (70%) rated their health as very good or good, one quarter as satisfactory and 5% as bad or very bad. Almost everybody (96%) thought that could do much/very much for their health.

The mean body mass index (BMI) was 21.7 kg/m^2 ^(min. 15.8, max. 32.3; SD: 3.16). According to the WHO's categories of obesity by BMI, 14% of the student were thin, 71% were in the normal range, 13% were preobese, and 2% obese.

### Mental health

Nearly one fifth (19%) of the students scored above the threshold (4 points) on the GHQ-12 indicating notable psychological distress. The proportion of those who suffered from psychological distress decreased during the study years and it was almost 1/3 lower among last year students than among freshmen (12.5 vs 35.3%).

The mean score for sense of coherence (SoC) was 60.1 (SD: 10.98, min. 31, max. 89). The mean score shows an increasing trend during the academic years, from 54.5 to 62.3 point.

As to social support, 59.5% of the students reported no lack of it, 23% lacked somewhat, and 17.5% severely lacked social support. The proportion of those who severely lacked social support is significantly higher among men than women (37.5% vs 12.7%; p: 0.003); 29% lacked social support from their student peers.

### Health behaviour

Nearly half (47%) of the students were non-smokers, 2% were former smokers, 23% were occasional smokers. More than one quarter (28%) smoked daily of whom 16% were heavy smokers using more than one package cigarette per day. Nearly three quarter (73%) of the smokers tried to give up smoking; 30% smoked less, and an equal proportion (30%) smoked for longer than 2 years, one fifth started smoking in the last 2 years. The mean age when students started smoking was 17 years.

Three quarter of the students drank alcohol, mainly occasionally. Only one person answered drinking every day; two students drank 3-4 times a week.

More than one-quarter (28%) had already tried some drugs, mostly marijuana and non-prescription narcotics and sedatives. The most common motivations to use drugs were curiosity (46%), to feel good (15%), and try to forget about problems (12%).

Majority (83%) of the students do some kind of physical activity for at least 10 min, 28% of them daily, 65.5% at least once a week.

Students had breakfast on average 5 times a week, 48% had breakfast every day and 4% never. More than half of them (54.5%) ate fruits and vegetables minimum one time per day, 26% consumed 2 or 3 times a week, and only one student did not eat fruits and vegetables in the past month. Nearly two third (63%) of the students used vegetable oil to cooking, and one third (31%) used vegetable oil and fat.

Almost one fifth (18%) of the respondents had never had sexual intercourse. All but 3 students used some kind of contraception: 47% condom and 41% contraceptive pills.

### Correlation analysis

Correlation analysis revealed significant correlation between social support and variables of mental health such as sense of coherence, as well as between social support and distress measured by the GHQ (Table 1). The negative correlation between social support and GHQ is explained by the fact that higher scores of social support reflect high support whereas low GHQ scores reveal good mental health. Significant correlation was found between social support and how much students can do for their health. In terms of social support and behaviour, significant inverse correlation was found between social support and use of sedative without prescription (correlation coefficient: -0.261, p: 0.002). Other variables of health status or behaviour were not related to social support (not shown).

**Table 1:** Correlation between social support and variables of mental health

Variables of mental health	Correlation coefficient	*P *value
Sense of coherence	0.306	*p *< 0.001

Psychological distress (GHQ)	−0.453	*p *< 0.001

Health locus of control (how much students can do for their health)	0.257	*p *= 0.002

Result of the correlation analysis between social support and variables for mental health

## Discussion

### Principal findings

The respondents were representative by study year, sex and age to all public health students who were in a 5-year training in Hungary (the Faculty providing the only such training in the country). Almost one-fifth of the students scored above the strict threshold on the GHQ indicating notable psychological distress. The proportion of those who suffered from psychological distress was almost 3 times higher among freshmen than among last year students. Since questions of the GHQ inquire about the preceding weeks, and our data were collected in the second part of the autumn semester, the exam period had no effect on the results. Nearly one fifth of the student severely lacked social support, and almost one third lacked social support from their student peers.

Correlation analysis, as expected, showed significant correlation between social support and variables for mental health, better mental health correlating with stronger social support. This finding is similar to our previous results observed among medical students [19], and to the result of a study on American college students according to which students with lower social support were more likely to experience mental health problems; however social support was measured by a different tool [29].

### Mental health and behaviour of public health students compared to other university students

In order to compare the health status and health behaviour of public health students in Hungary to their student peers in other countries, we compared our data with data from the literature.

Subjective health of students from universities in Germany, Bulgaria, and Poland was good or very good in 81%, which is higher than in our sample (70%). The proportion of preobese or obese students was 12%, 3% lower than in our study [30]. The mean BMI among Spanish university students was lower in both sexes than in Hungarian students (males: 22.9 vs 24.0, females: 20.6 vs 21.2) [31].

One study of Finnish students found the mean score for sense of coherence to be 62.6 and the mean score for psychological distress 24.0 at graduation [32], both close to the mean score found in our study (the mean score for psychological distress using the same scale was 24.7 in the present survey).

The prevalence of non-smoking was higher among students from universities in Germany, Bulgaria, and Poland (65% vs 47%) [30]. A study of public health and emergency medicine students at the Faculty of Health Sciences of the Medical University of Lodz found the ratio of the smoking female students to be 34% and smoking male students 46% [33]. The proportion of female smokers was higher (51%) in our study, but that of the male smokers was similar (45%). However, the proportion of daily smokers was 2 times higher in Turkish students (59% vs 28%) [34].

The prevalence of teetotaler students from universities in seven European countries varied in males between 8% and 73%, in average 49%, in females between 16% and 88%, in average 34% [35]. This was 25% in our sample in both sexes, lower than the average of the seven European countries.

### Mental health and behaviour of public health students compared to peers

In order to interpret our data more precisely we have compared our results with data of the Hungarian National Health Interview Survey 2003 in same age group in which identical tools were used for the variables shown in Tables 2 and 3. Because of the significant difference in the proportion of sexes in our sample compared to that of the general population, data were stratified for males and females.

**Table 2:** Comparison of the health status of male public health students and their peers

		Public health students (19-25 years males)	National health interview survey 2003 (19-25 years males)	*p *value
Perceived health is very good or good		75%	83%	0.341

Can do much/very much for their health		96%	90%	0.376

BMI (mean)		24.04	23.89	0.841

Above the threshold on GHQ		12.5%	8.34%	0.484

Severe lack of social support		37.5%	9.87%	**<0.001**

Nutrition	Breakfast every day	50%	66%	0.119

	Daily fruit & vegetable consumption	54%	41%	0.198

Smoking	Non-smokers	54.5%	41%	0.202

	Daily smokers	32%	46%	0.188

Alcohol consumption		75%	71%	0.698

Comparison of some health measures and health behaviour of male public health students and males of the same age group (19-25 years) of the Hungarian National Health Interview Survey 2003. Indicators significantly worse in public health students are highlighted in bold.

**Table 3:** Comparison of the health status of female public health students and their peers

		Public health students (19-25 years females)	National health interview survey 2003 (19-25 years females)	*p *value
Perceived health is very good or good		68%	77%	0.079

Can do much/very much for their health		97%	88%	*0.005*

BMI (mean)		21.17	21.89	0.078

Above the threshold on GHQ		19.83%	12.20%	**0.044**

Severely lack of social support		12.71%	12.29%	0.907

Nutrition	Breakfast every day	48%	61%	**0.013**

	Daily fruit & vegetable consumption	55%	56%	0.906

Smoking	Non-smokers	46%	51%	0.373

	Daily smokers	27%	31%	0.402

Alcohol consumption		75%	44%	**<0.001**

Comparison of some health measures and health behaviour of female public health students and females of the same age group (19-25 years) of the Hungarian National Health Interview Survey 2003. Indicators significantly worse in public health students are highlighted in bold; indicators significantly better in public health students are highlighted in italic.

The mean age of the chosen age group of the general population (19-25 years) was 22 years, 49% of them male.

There was no significant difference between the health status and behaviour of male public health students and their non-student peers (Table 2). However, the proportion of those male students who severely lack social support was nearly four times higher (*p *< 0.001).

Nearly all of the female public health students (Table 3) thought that they can do much/very much for their health, and this proportion is significantly higher (p: 0.005). Female students had breakfast less frequently (p: 0.013) and drank alcohol nearly 2 times more (*p *< 0.001). Significantly more students scored above threshold in terms of psychological distress compared to their peers (p: 0.044).

Comparison of our health behaviour data to the same age-group of the general population revealed that the proportion of alcohol drinkers is higher among female students. In terms of mental health status, female students scored worse, while lack of social support was a greater problem for male students.

These results are in line with the result of a survey among British students in higher education who scored significantly worse than their peers in the local population on SF-36 dimensions. The authors concluded that the health of students is poor relative to that of their peers, and that their emotional health is more of a problem than their physical health [36].

### Strengths and limitations

An advantage of the present survey is that the surveyed population, in spite of being relatively small, gave a high response rate, and respondents represented students by study year, sex and age in the Faculty. Though the respondents are not representative either for all Hungarian students in higher education or for students of University of Debrecen but reasonably cautious conclusions can be drawn for all public health students in the country given that the study aimed at 51% of all full-time public health students in Hungary. The proportion of women among all public health students in the country was 86%, not significantly different from the proportion of women in our study (p: 0.468).

The cross-sectional design of the study does not allow conclusions on a causal relationship. Our aim was to investigate the association between social support and the parameters for mental health and health behaviour in public health students. Potential sources of bias in questionnaire surveys may arise from the respondents not answering honestly, or not remembering for their particular behaviour. This type of bias was probably not higher than in similar studies using standardized scales so our results are reasonably comparable with the results of other studies.

The timing of data collection about mental health is a critical point in case of university students because their stress level can change during the academic year. A potential source of bias might be due to the collection of data close to the exam period. In order to reduce this type of bias, data were collected in the mid-term of the autumn semester. The timing draws further attention to the unfavourable mental health status of university students in contrast to the findings of the Eurobarometer mental health survey 2010. This survey found that those who experience positive mental health status tend to be 15-24 year-olds and students [37].

### Conclusions: meaning of the study and future research

Psychological morbidity occurs significantly more frequently in Hungarian female students of public health compared to their peers, while the mental health of male public health students was very similar to their non-student peers except for the notably more frequent lack of social support. Social support strongly and positively correlates with better mental health among public health students. Our results highlight the importance of further, preferably longitudinal research on the mental health and behaviour of public health students in light of their status as future role models of health among their clients. Training institutions should enhance training to improve coping skills for all students or increase social support (or both) as potentially amenable determinants of mental health during higher education, and/or should provide more and better targeted services for those with highest risk not only in order to improve students' mental health but also to increase their future credibility as professionals who walk the talk of public health and talk the walk of its paths.

## Authors' contributions

KK and ÁR developed the concept for this paper. BÉ directed the field work, was responsible for data management, data analyses and the statistical procedures and tests, interpreted the results, conducted the literature search, and interacted with co-authors in subsequent drafts of the paper. KK was also responsible for assisting with data analysis, interpretation of results, and writing of the paper at all stages. ÁR contributed towards writing of the paper at all stages. All authors read and approved the final manuscript.

## Competing interests

The authors declare that they have no competing interests.

## Pre-publication history

The pre-publication history for this paper can be accessed here:

http://www.biomedcentral.com/1471-2458/11/871/prepub
